# More staff = better quality of life for people with dementia? results of a secondary data analysis in German shared-housing arrangements

**DOI:** 10.1186/s13584-019-0295-7

**Published:** 2019-02-20

**Authors:** Johannes Gräske, Annika Schmidt, Karin Wolf-Ostermann

**Affiliations:** 1 School of Social Sciences, Department of Health and Nursing, HTW Saar, Saarbrucken, Germany; 2 Health Services Research in Nursing Sciences, Institute of Public Health and Nursing Research, University of Bremen, Bremen, Germany

**Keywords:** Dementia care, Quality of life, Shared-housing arrangements, Staffing

## Abstract

**Background:**

Shared-housing arrangements (SHAs) in Germany are an alternative care arrangement for people with dementia. They are disconnected from traditional nursing homes and are often situated in ordinary apartment buildings. Community health care providers serve persons with dementia in SHAs, and there is no official regulation regarding the staff-resident ratio. The association between the staff-resident ratio and the quality of life (QoL) of persons with dementia has not yet been investigated in SHAs.

**Method:**

A cross-sectional study was performed in SHAs in Berlin, Germany, using ANCOVA models to analyse whether residents’ QoL (QUALIDEM), as assessed by staff in SHAs, can be explained by the staff-resident ratio, adjusted for residents’ sex, age, length of stay, challenging behaviour (CMAI), cognitive impairment (GDS) and level of care dependency according to the German statutory health care insurance.

**Results:**

In this study, 58 SHAs with 396 residents (mean age 78.4 years, 69.4% female) participated. The staff-resident ratio was 0.2 and 0.6 for registered nurses and certified nursing assistants, respectively. Associations with QoL were found predominantly for challenging behaviour and cognitive impairment. The analysis showed that there was no significant effect of the total staff-resident ratio (*p* > 0.05) in explaining the variation in residents’ QoL (total and subdomains). In general, the proportion of explained variance was weak (R^2^ <  0.216).

**Conclusions:**

The present study did not show a significant association between staffing and residents’ QoL in SHAs. However, further investigation is required regarding the direct interaction between staff and residents. A main focus should be to educate users about the benefits and disadvantages of shared-housing arrangements.

## Background

In 2050, the number of persons with dementia is estimated to be 115 million worldwide [1]. In Germany, the number will rise from approximately 1.1 million in 2008 to 3.3 million in 2050 [2]. Dementia causes challenges in home care for persons with dementia and their family members, often resulting in patients being relocated into nursing homes [3]. However, nursing homes are frequently criticised for their task-oriented care provision; therefore, the Organisation for Economic Co-operation and Development (OECD) suggests that long-term care provision should be organised to be as homelike as possible [4]. In line with this recommendation, group living developed in Sweden in the 1980s [5]. Subsequently, comparable models were established all over the world, e.g., Green Houses in the USA, group homes in Japan, small-scale living arrangements in the Netherlands, and German shared-housing arrangements (SHAs) [6, 7]. These arrangements are different from traditional care, tending towards a small and homelike arrangement, with a person-centred care approach respecting the residents’ needs and choices [7]. While in traditional care arrangements, daily routines are organised around nursing tasks, in small-scale living facilities, routines include household chores (e.g., cooking, baking) to promote the principle of normal living. As an example, the German concept of SHAs will be described in more detail.

### Shared-housing arrangements

The first German SHA was founded in Berlin in 1995 by family members of persons with dementia [8]. The main motivation was to seek alternative concepts of care and support to improve the self-determination of persons with dementia. Since then, the number of SHAs has increased, and Wolf-Ostermann et al. [6] identified more than 1400 SHAs all over Germany in 2012. Rothgang et al. counted 3891 SHA in Germany in 2017 [9]. In 2017, Klie et al. estimated the number of SHAs to be more than 3100 [10], 690 of which were located in Berlin. Residents of SHAs (typically 6–8) are predominantly female, are approximately 80 years old and show advanced care dependency. Most of the residents have dementia, and even when the level of care dependency increases or the residents are moribund, they usually stay within the SHA [11]. SHAs are separate from traditional nursing homes, often situated in ordinary apartment buildings, and persons with dementia in SHAs are served by community health care providers [11].

### Staff in SHAs

Since SHAs in Germany are mostly a form of private living, they are not bound to the same legal restrictions as nursing homes concerning care and support. An official regulation for SHAs only exists in few federal states; for example, in Berlin, at least one certified nursing assistant (CNA) must be present 24 h a day and 7 days a week. However, there is an ongoing discussion throughout all states to increase staff numbers. Therefore, residents, their family members or legal representatives must order more staff if they feel that the number of staff is insufficient in the SHA. On the other hand, health care providers can suggest that more staff are necessary. However, residents or their representatives make the final decision. In addition, they must pay an additional fee, because greater availability of staff allows for the provision of more health care services is more expensive than less staff. Within SHAs, registered nurses (RNs), CNAs and housekeepers work together to provide care and support. A study in 2009 showed, on average, 6.9 employees (full-time equivalent; RN, CNA and housekeeper) per SHA, which equates to a staff-resident ratio of 1.3 [12]. Compared to the US model Green Houses [13], in German SHAs, the caregivers are predominantly CNAs. In 2009, the staff-resident ratio in the SHAs was 0.7 for CNAs, while the ratio for RNs was 0.4 and for housekeepers was 0.2 [12]. The organisation within the SHA follows the principle of task sharing, with RNs usually coming into the SHA to perform principal nursing tasks such as injections or the administration of drugs. Additionally, the housekeepers are not present throughout the whole day, often only present to prepare meals, to assist with feeding and to perform household chores [11]. Compared to special-care units (SCUs) in nursing homes, Wolf-Ostermann et al. [14] reported a better total staff ratio for SHAs, but due to official regulations, the staff ratio restricted to RNs is better in SCUs.

There is a lack of research regarding the impact of a better staff-resident ratio in dementia care on patient-related outcomes such as quality of life. In her 2015 study, Chenoweth found that a better staff-resident ratio in dementia care is associated with a better quality of care [15]. In their systematic review, Xu et al. [16] found inconsistent evidence regarding whether a better staff-resident ratio in nursing homes improves residents’ quality of life (QoL). For alternative forms of housing such as SHAs, there is no evidence that a better staff-resident ratio is associated with a better quality of life; therefore, the present study aims to investigate this research gap. The main research questions are as follows:

1. What is the average staff-resident ratio (total staff, RNs, CNAs, housekeepers) in SHAs?
2. Is there an association between the staff-resident ratio and residents’ QoL in SHAs?

## Methods

To address these questions, a cross-sectional study was performed as part of the larger WGQual study [17, 18]. Written standardised questionnaires were sent to community health care providers of all registered SHAs in Berlin, Germany. When questionnaires were not returned, all providers received a polite reminder via phone. Head nurses and social workers completed the questionnaire together. The questionnaire asked about actual data on present staff in the SHAs in the month before data collection. Furthermore, data were collected on all residents’ socio-demographics (age, sex), level of care dependency according to the German statutory health care insurance (0: no physical need, I, II and III: requires at least 90 min, 3 h and 5 h, respectively, of care and help per day) [19], QoL (QUALIDEM), challenging behaviour (CMAI) and cognitive impairment (GDS).

### Staff

Given the previously described task sharing, it was necessary to assess the exact amount of time that each staff member was actively present in the SHAs. To avoid outliers, data were assessed for a whole month for total staff as well as for RNs, CNAs and housekeepers separately. Subsequently, the average full-time equivalents were calculated for each group per day, and the average total amount of staff per day was also calculated.

### Quality of life

The QUALIDEM [20, 21] was used to assess residents’ QoL. It is a proxy-rated dementia-specific instrument that is considered appropriate to assess residents’ QoL in SHAs [22, 23]. The instrument comprises 37 items (rated on a Likert scale from *never* to *daily*) with nine subdomains: *care relationship* (seven items), *positive affect* (six items), *negative affect* (three items), *restless tense behaviour* (three items), *positive self-image* (three items), *social relations* (six items), *social isolation* (three items), *feeling at home* (four items), *having something to do* (two items). However, for people with very severe dementia, only six subscales (*care relationship*, *positive affect*, *negative affect*, *restless tense behaviour*, *social relations*, *social isolation*) are relevant [24]. A *total QoL* score was calculated by summing all applicable items. For better comparability, all sum scores (subdomains and the *total QoL)* were linearly adapted to a scale ranging from 0 to 100, with higher scores indicating a better QoL. The QUALIDEM shows good validity and reliability [22, 25].

### Challenging behaviour

The Cohen-Mansfield Agitation Inventory (CMAI) [26] was used to assess residents’ challenging behaviour. This instrument includes 29 behaviours rated on a 7-point Likert scale (from *never* to *a few times in an hour*). The subscales indicate the occurrence of *aggressive behaviour*, *physically non-aggressive behaviour* or *verbally agitated behaviour* [27]. Additionally, whether a resident showed at least one challenging behaviour was calculated. The CMAI shows good values of reliability and validity.

### Severity of dementia

Cognitive decline was assessed by the Global Deterioration Scale (GDS) [28], which includes seven different stages ranging from *no cognitive decline* to *very severe cognitive decline*. In the present study, there were only a few persons with dementia with a GDS of less than six (*severe cognitive decline*); therefore, the categories of five and below were combined into one category.

### Data analysis

The data were described by typical parameters, e.g., means and standard deviations (SDs), and Pearson as well as Spearman correlations were performed for metric data. The effect of staff-resident ratio on residents’ QoL was analysed using ANCOVA models, including all subdomains of the QUALIDEM and the *total QoL*. Influencing factors were taken into account regarding residents’ sex, age, GDS, occurrence of at least one need-driven behaviour (CMAI) and level of care. Additionally, the staff-resident ratios for total staff were considered. Due to the multicollinearity (see Results section) between the ratios for RNs, CNAs, housekeepers and total staff, only the total staff ratio was included in the ANCOVA analyses. Interactions between independent variables were not modelled because of the small number of participants. Before further analyses were conducted, statistical model assumptions were examined. Statistical significance was specified as *p* ≤ 0.05. Post hoc power calculations were performed using G*Power 3.1.9.3.

## Results

The response rate was 12.8% of all registered SHAs in Berlin and 15.2% of all estimated residents in Berlin. In total, 58 SHAs and 396 residents contributed to the study, which is equivalent to a mean number of 6.9 (SD 2.2) residents per SHA.

### Sample characteristics

Table 1 displays the residents’ socio-demographic characteristics, severity of dementia, challenging behaviour and QoL scores. Residents in SHAs are, on average, 78.4 (SD 11.1) years old and predominantly female (69.4%). All residents somehow show a need for care, and approximately 70% of them show a high level of care dependency. The mean length of stay in the SHAs was almost 3 years, with 71% of all participating residents having a medical diagnosis of dementia, predominantly in the severe stage (GDS 6: 42.2% and GDS 7: 38.4%). Nearly 60% of all residents showed at least one challenging behaviour, while their QoL (*total QoL* and subdomains) was moderate to good. An in-depth sample description of these data has been published previously [29].

**Table 1:** Characteristics of study sample

	Residents (*n* = 396)
Age in years; mean (SD; Min-Max)	78.4 (11.1; 45–102)
Women in % (n)	69.4 (275)
Time being in the SHA in years; mean (SD)	2.7 (2.3)
Medical diagnosis of dementia in % (n)	71.0 (281)
Cognitive decline (GDS; 0–7) in % (*n*)	
≤ 5 (moderately severe cognitive decline)	15.9 (63)
6 (severe cognitive decline)	42.2 (167)
7 (very severe cognitive decline)	38.4 (152)
Challenging behavior (CMAI) in % (*n*)	
Physically nonaggressive behavior	34.3 (136)
Verbally agitated behavior	37.9 (150)
Aggressive behavior	15.9 (63)
At least one challenging behavior	57.8 (229)
Level of care dependency in % (*n*; 0-III) ^a^	
0	7.3 (27)
Level I	21.7 (80)
Level II	49.3 (182)
Level III	21.7 (80)
Quality of Life (QUALIDEM; 0–100); mean (SD; Min-Max)	
Total QoL	69.5 (14.1; 24,5-97,2)
Care relationship	72.1 (21.0; 4,8–100)
Positive affect	73.1 (23.6; 0–100)
Negative affect	71.6 (23.7; 0–100)
Restless tense behavior	63.2 (30.1; 0–100)
Positive self-image^b^	75.5 (23.1; 0–100)
Social relationship	68.8 (21.9; 0–100)
Social isolation	69.4 (22.4; 0–100)
Feeling at home^b^	80.7 (18.2; 16,7–100)
Having something to do^b^	56.2 (26.5; 0–100)

^a^Care level determined by the German long-term care insurance, ^b^not applied for Global Deterioration Scale 7; underlined values are most beneficial; *SD* standard deviation, *Min* minimum, *max* maximum, *GDS* Global Deterioration Scale, *CMAI* Cohen-Mansfield Agitation Inventory

### Staffing in SHAs

On average, 7.1 (SD 5.2) staff members were actively present within the SHA per day, resulting in a staff-resident ratio of 0.9 (SD 0.2) (see Table 2). The professional group with the highest number of staff members present in SHAs was CNAs, with an average of 4.7 (SD 3.9) of them present, or 0.6 (SD 0.5) CNAs per resident present per day. RNs and housekeepers were equally present (1.3 for each group, or 0.2 of each group per resident). There was no correlation between the severity of dementia (GDS) and any staff-resident ratios (all Spearman rho *p* > 0.05). The staff-resident ratio regarding the total staff correlated positively with the ratio of RNs (Pearson r = 0.365; *p* <  0.001), CNAs (Pearson r = 0.906; *p* <  0.001) and housekeepers (Pearson *r* = 0.695; *p* <  0.001). Furthermore, there was a positive correlation between the ratio of CNAs and housekeepers (Pearson r = 0.490; *p* <  0.001).

**Table 2:** Staffing in Shared-housing Arrangements

	Absolut mean (SD)	Staff-resident ratio mean (SD)
Total staff	7.1 (5.2)	0.9 (0.2)
Registered Nurses	1.3 (1.3)	0.2 (0.2)
Certified Nursing Assistance	4.7 (3.9)	0.6 (0.5)
Housekeeping	1.3 (1.5)	0.2 (0.2)

### Associations between staffing and residents’ QoL

Weak negative correlations were found between the total staff-resident ratio for *total QoL* (Pearson *r* = − 0.113; *p* = 0.029), QUALIDEM *negative affect* (Pearson r = − 0.107; *p* = 0.040), QUALIDEM *restless tense behaviour* (Pearson *r* = − 0.166; *p* <  0.001) and QUALIDEM *social isolation* (Pearson r = − 0.123; *p* = 0.018). All other subdomains showed no significant correlation with the amount of staff within the SHAs.

In the ANCOVA analyses, all subdomains of the QUALIDEM (except positive affect) showed significant effects of the independent variables (*p* <  0.05). For the QUALIDEM, the *total QoL score* and *restless tense behaviour* of older people and people without challenging behaviour or a lower severity of dementia showed a better QoL (see Table 3). People without challenging behaviour and a lower severity of dementia had a better QoL in terms of *positive self-image* and *social isolation* (see Table 3). For the subscale *care relationship,* female residents, as well as those without challenging behaviour and older persons, had a better QoL. Men and persons without challenging behaviour showed a better QoL in terms of *negative affect*. Better QoL was observed for *social relationships* among persons without challenging behaviour and for *having something to do* among persons with better cognitive functioning.

**Table 3:** Analysis of Variance Quality of Life (QUALIDEM)

Dependent Variable	*p*-Value Model	R^2^	Independent (Co-)Variable	*p*-Value Variable	Estimate	95% CI
QUALIDEM: *total Score*	**< 0.001**	**0.216**	**Intercept**	**< 0.001**	**37.99**	**24.65:51.34**
			Sex: female^a^	0.253	1.84	− 1.32;5.01
			**At least 1 challenging behaviour (CMAI)** ^**b**^	**< 0.001**	**−10.40**	**− 13.22;- 7.58**
			**Cognitive decline (GDS)** ^**c**^			
			**≤ 5**	**0.008**	**6.75**	**1.77;11.73**
			6	0.195	2.29	−1.18;5.76
			Level of care dependency^d^			
			0	0.111	6.06	−1.40;13.52
			I	0.054	5.17	−0.09;10.43
			II	0.737	−0.68	−4.63;3.28
			Age*	**0.001**	**0.27**	**0.12;0.42**
			Length of atay^e,^*	0.230	0.39	−0.245;1.02
			Ratio total-staff per resident*	0.804	0.25	−1.75;2.25
QUALIDEM: *care relationship*	**< 0.001**	**0.163**	**Intercept**	**< 0.001**	**46.96**	**26.08;67.85**
			**Sex: female** ^**a**^	**0.043**	**5.11**	**0.154;10.07**
			**At least 1 challenging behaviour (CMAI)** ^**b**^	**< 0.001**	**−14.51**	**− 18.93;-10.10**
			Cognitive decline (GDS)^c^			
			≤ 5	0.188	5.23	−2.57;13.02
			6	0.140	4.08	−1.35;9.51
			Level of care dependency^d^			
			0	0.296	−6.21	−17.88;5.47
			I	0.239	−4.93	−13.17;3.30
			**II**	**0.017**	**−7.57**	**−13.76;-1.39**
			Age*	**0.032**	**0.26**	**0.02;0.50**
			Length of stay^e,^*	0.281	−0.54	−1.53;0.47
			Ratio total-staff per resident *	0.442	−1.23	−4.36;1.91
QUALIDEM: *positive affect*	0.133	0.045	**Intercept**	**0.001**	**40.61**	**16.08;65.13**
			Sex: female^a^	0.140	4.38	− 1.44;10.20
			at least 1 challenging behaviour (CMAI)^b^	0.123	−4.08	−9.26;1.11
			Cognitive decline (GDS)^c^			
			≤ 5	0.147	6.76	−2.40;18.39
			6	0.070	5.89	−0.49;12.27
			Level of care dependency^d^			
			0	0.502	4.68	−9.03;18.39
			I	0.688	1.98	−7.69;11.65
			II	0.630	−1.78	−9.04;5.48
			Age*	0.053	0.27	−0.01;0.55
			Length of stay^e,^*	0.680	0.24	−0.92;1.40
			Ratio total-staff per resident *	0.305	1.92	−1.76;5.59
QUALIDEM: *negative affect*	**< 0.001**	**0.149**	**Intercept**	**< 0.001**	**57.00**	**33.32;80.69**
			**Sex: female** ^**a**^	**0.006**	**−7.95**	**−13.57;-2.33**
			**At least 1 challenging behaviour (CMAI)** ^**b**^	**< 0.001**	**−12.25**	**−17.26;-7.24**
			Cognitive decline (GDS)^c^			
			≤ 5	0.211	5.63	−3.21;14.47
			6	0.169	−4.31	−10.47;1.85
			Level of care dependency^d^			
			0	0.222	8.23	−5.01;21.47
			I	0.077	8.43	−0.91;17.76
			II	0.403	2.99	−4.03;10.00
			Age*	0.360	0.125	−0.14;0.39
			Length of stay^e,^*	0.112	0.91	−0.21;2.03
			Ratio total-staff per resident *	0.937	−0.143	−3.69;3.41
QUALIDEM: *restless tense behavior*	**< 0.001**	**0.186**	Intercept	0.899	1.85	−26.86;30.56
			Sex: female^a^	0.592	−1.86	−8.67;4.96
			**At least 1 challenging behaviour (CMAI)** ^**b**^	**< 0.001**	**−13.99**	**− 20.06;-7.92**
			**Cognitive decline (GDS)** ^**c**^			
			**≤ 5**	**0.009**	**14.24**	**3.53;24.96**
			**6**	**0.001**	**13.30**	**5.84;20.77**
			Level of care dependency^d^			
			0	0.111	13.03	−3.02;29.08
			I	0.013	14.39	− 3.07;25.71
			II	0.094	7.26	−1.25;15.76
			Age*	**0.001**	**0.54**	**0.21;0.86**
			Length of stay^e,^*	0.259	0.78	−0.58;2.14
			Ratio total-staff per resident *	0.182	−2.93	−7.23;1.37
QUALIDEM: *positive self-image*	**0.001**	**0.145**	**Intercept**	**0.014**	**47.02**	**9.63;84.41**
			Sex: female^a^	0.106	−5.56	−13.19;-1.28
			**At least 1 challenging behaviour (CMAI)** ^**b**^	**0.005**	**−9.42**	**−2.82;-16.02;**
			**Cognitive decline (GDS)** ^**c**^			
			**≤ 5**	**0.030**	**8.79**	**0.86;16.72**
			Level of care dependency^d^			
			0	0.349	9.62	−10.61;29.86
			I	0.845	−1.75	− 19.30;15.81
			II	0.549	−5.00	−21.41;11.42
			Age*	0.057	0.36	−0.01;0.73
			Length of stay^e,^*	0.454	−0.60	−2.17;0.98
			Ratio total-staff per resident *	0.391	2.21	−2.85;7.27
QUALIDEM: *social relationship*	**< 0.001**	**0.176**	**Intercept**	**< 0.001**	**42.58**	**20.75;64.40**
			Sex: female^a^	0.298	2.74	−2.44;7.92
			**At least 1 challenging behaviour (CMAI)** ^**b**^	**< 0.001**	**−16.38**	**−21.00;11.77**
			Cognitive decline (GDS)^c^			
			≤ 5	0.084	7.17	−0.98;15.31
			6	0.182	−3.86	−1.82;19.53
			Level of care^d^			
			0	0.219	7.63	−4.57;19.84
			I	0.297	4.57	−4.04;13.17
			II	0.565	−1.89	−8.36;4.57
			Age*	0.158	0.178	− 0.07;0.43
			Length of stay^e,^*	0.456	0.39	−0.64;1.42
			Ratio total-staff per resident *	0.688	−0.668	−3.94;2.60
QUALIDEM: *social isolation*	0.148	0.176	**Intercept**	**< 0.001**	**42.58**	**20.75;64.40**
			Sex: female^a^	0.298	2.74	−2.44;7.92
			**at least 1 challenging behaviour (CMAI)** ^**b**^	**< 0.001**	**−16.38**	**−11.77;-0.80**
			**Cognitive decline (GDS)** ^**c**^			
			**≤ 5**	**0.084**	**7.165**	**−0.98;15.31**
			6	0.182	3.86	−1.82;9.53
			Level of care dependency^d^			
			0	0.219	7.63	−4.57;19.84
			I	0.297	4.57	−4.04;13.17
			II	0.565	−1.89	−8.36;4.57
			Age*	0.158	0.178	−0.07;0.43
			Length of stay^e,^*	0.456	0.39	−0.64;1.42
			Ratio total-staff per resident *	0.688	−0.668	−3.94;2.60
QUALIDEM: *feeling at home*	0.148	0.070	**Intercept**	**< 0.001**	**69.33**	**38.27;100.39**
			Sex: female^a^	0.662	−1.33	−7.34;4.68
			**at least 1 challenging behaviour (CMAI)** ^**b**^	**0.025**	**−6.28**	**−11.77;0.80**
			**Cognitive decline (GDS)** ^**f**^			
			≤ 5	0.276	−3.65	−10.23;2.94
			Level of care dependency^d^			
			0	0.970	0.32	−16.49;17.13
			I	0.478	−5.26	−19.84;9.33
			II	0.802	−1.73	− 15.37;11.90
			Age*	0.407	0.129	−0.18;0.44
			Length of stay^e,^*	0.176	0.90	−0.41;2.21
			Ratio total-staff per resident *	1.00	−0.01	−4.20;4.20
QUALIDEM: *having something to do*	**< 0.001**	**0.157**	**Intercept**	**0.041**	**43.81**	**1.87;85.76**
			Sex: female^a^	0.483	2.89	−5.23;11.00
			at least 1 challenging behaviour (CMAI)^b^	0.088	−6.44	−13.84;0.97
			**Cognitive decline (GDS)** ^**f**^			
			**≤ 5**	**0.004**	**13.01**	**4.11;21.90**
			Level of caredependency^d^			
			0	0.512	7.56	−15.14;30.26
			I	0.328	9.80	−9.90;29.49
			II	0.709	−3.49	−21.90;14.93
			Age*	0.987	0.210	−0.42;0.41
			Length of stay^e,^*	0.439	−0.69	−2.46;1.07
			Ratio total-staff per Resident *	0.152	4.14	−1.54;9.82

^a^compared to male; ^b^compared to no challenging behaviour; ^c^compared to GDS 7; ^d^compared to care dependency level III ^e^in the shared-housing arrangement; ^f^compared to GDS 6; *CMAI* Cohen-Mansfield-Agitation Inventory, *GDS* Global Deterioration Scale, *RN* Registered nurse, *CNA* Certified nursing assistant; *Co-Variables

The analyses did not yield a significant effect of the total staff-resident ratio (*p* > 0.05) on residents’ QoL (total and subdomains). Generally, the proportion of the explained variance was weak (R^2^ <  0.216). A post hoc power calculation showed a power of 0.9866.

## Discussion

Because caring for persons with dementia will become more challenging in the future, the identification of approaches to improve their QoL is important. Therefore, the present paper aimed to identify the impact of the staff-resident ratio in SHAs on residents’ QoL. The characteristics (socio-demographics as well as challenging behaviour) of the included sample represent typical SHA residents [18, 30]. Additionally, the level of care dependency of the persons with dementia was comparable to that shown in earlier studies. In 2009, residents of SHAs were predominantly graded in care level II (49.8%), which is similar to the findings in the present study (49.3%); therefore, it was assumed that there was no sample bias.

### Staffing

The identified number of staff present in the SHAs was also comparable to that found in previous studies [12, 14], with only the ratio of RNs being slightly lower. RNs are usually only present in SHAs for administering medications or performing other tasks that must be performed by an RN. It is surprising that the number of staff in the present study was not equivalent to that in previous studies.

### Quality of life

The QoL scores indicated moderate to good QoL of the persons with dementia, with the highest scores reported for *positive self-image* and *feeling at home*. The lowest QoL was found for the subscales *restless tense behaviour* and *having something to do.* Generally, the scores were comparable to those from another study in an SHA setting [30] as well as a study of German nursing homes [31].

### Associations with quality of life

QoL assessments are proxy measurements performed by nurses working in the SHA. The analyses showed a strong negative impact on proxy-rated QoL for showing challenging behaviour and a more severe level of dementia (see also [29]); both of these findings have been identified in the literature. In their reviews, Banerjee et al. and Beerens et al. found strong negative associations for both aspects with proxy-rated QoL [32, 33]. Additionally, the present study found heterogeneous results for age and sex. Only a few subscales showed a significant association with the independent variables considered; sex especially yielded different results, and females showed a higher QoL than men or vice versa on some scales. Although the power of the presented analysis was sufficient, no QUALIDEM scale showed a significant association with the staff-resident ratio. This finding is surprising, given that the subscale *care relationship* is linked to the aspect of staffing. It could have been expected that this subdomain is affected by different staff-resident ratios; however, the results are in line with those of Xu et al. [16], who also found no convincing associations between staffing and the QoL of residents in a nursing home. It is obvious that people with higher care dependency require a better staff-resident ratio, but care dependency or impairment in activities of daily living were negatively associated with proxy-rated QoL [32, 33]. Therefore, a better total staff-resident ratio may compensate for the greater impairment. Nonetheless, the mean length of time a staff member has been caring for a specific resident was not evaluated in the present study. A high staff ratio within the SHA does not necessarily mean that the staff sees an individual more often. Therefore, every individual resident does not necessarily benefit from a higher ratio, thus this not means a higher quality of care. Future studies should investigate whether staff members’ longer time of care for a specific resident improves the QoL of persons with dementia and the quality of care provided by nurses. Additional information that is needed in similar studies is the social support provided by family members and/or volunteers.

### Implications

The findings showed that residents of SHAs are very unwell in terms of dementia severity and challenging behaviour symptoms, similar to findings reported in other European, North American and Australasian studies of similar populations. Despite no statistical relationship being detected between the staffing-resident ratio and quality of life, it is nonetheless possible that such an association exists. Persons with (severe) dementia must manage their immense fundamental care needs and satisfy their basic human needs. Person-centred care is regarded as a promising approach to provide tailored care to the most pertinent needs of the recipient and family members. In this case, needs are associated with cognitive decline as well as with symptoms of challenging behaviour and management from staff. In a diverse society, different approaches, especially in long-term care, are essential in person-centred care. In addition to the availability of SHAs alone, health politicians, health care insurances, non-government organisations (e.g., Alzheimer societies) and educators play a major role in informing potential users and their family members of opportunities to live in SHAs. A main focus should be to educate users about the benefits and disadvantages of SHA. Maintaining good QoL while the disease is progressing is a major goal in dementia care. Therefore, it will be of great benefit to investigate in more detail how QoL can be improved in care settings such as SHAs. This information could ensure that nurses are educated adequately and are enabled to develop QoL promotion strategies, concepts and techniques. Stakeholders are requested to create an environment where enough well-educated staff work together with family members. Finally, the scientific evaluation of the health care provided for the vulnerable population of persons with dementia is necessary.

## Conclusion

This is the first report of the impact of the staff-resident ratio on residents’ QoL in German SHAs. The SHA residents in this study showed typical characteristics of persons with dementia; however, there was no significant effect of the staff-resident ratio in German SHAs, explaining the variation in residents’ QoL as measured by the QUALIDEM but also the effects associated with cognitive impairment and challenging behaviour. Further investigation, for example, of the direct interaction between staff and residents, would provide a more in-depth insight into this issue of the impact of staff on residents’ QoL in dementia care.

### Limitations

This study has some limitations that must be stated before generalising these findings. The sample size was relatively large, but participants were only recruited from Berlin, Germany, so the transferability of the findings to rural areas or to different urban areas is questionable. Currently, a wider discussion about the level of agreement between self- and proxy ratings in QoL measurements is ongoing [34, 35]. Furthermore, staff characteristics, such as burn-out and satisfaction with life, may also influence the proxy ratings of residents’ QoL [36]. For practical reasons, these phenomena were not taken into account in the present study, which may have influenced the study findings. Finally, nurses, as care providers, rated the QoL of residents and therefore evaluated their own work. This aspect of the study might influence the ratings (e.g., *feeling at home* is rated as one of the highest QoL subscales).

## Abbreviations

CMAI: Cohen-Mansfield Agitation Inventory
CNA: Certified nursing assistant
GDS: Global Deterioration Scale
OECD: Organisation for Economic Co-operation and Development
QoL: Quality of life
RN: Registered nurse
SCU: Special-care unit
SD: Standard deviation
SHA: Shared-housing arrangement
